# Vasectomy and Eugenics

**Published:** 1911-07

**Authors:** 


### Vasectomy and Eugenics.

There are never lacking supporters of reform movements whose enthusiasm carries them even beyond that of Artemus Ward, who professed willingness to sacrifice all of his wife's relatives in the war, and those are in this class who expect wonderful eugenic results to follow vasectomy upon idiots, the feeble-minded, the incurable insane and habitual criminals; and it will be only a short step in advance to extend the sterilization to girls and women in the same classes.

There are no inalienable rights, not even that of life itself, and governments have in all ages asserted the right to self-preservation. There are good reasons why organized society should insist, under wise safeguards, upon the compulsory sterilization of idiots and feeble-minded persons, and there are equally good reasons why sterilization should not be compulsory among the insane and habitual criminals.

The only evil results which follow sterilization in human beings are physical and moral. Vasectomy has been performed upon several hundred individuals in the State of Indiana. Observations there indicate that the operation is not followed by any bad effects physically upon the growth or development of the individuals subjected to it, and the care of such wards of the State is immensely simplified by it. It very greatly diminishes the practice of masturbation and renders procreation impossible in those subjected to it.

The argument in favor of vasectomy in males applies with equal or greater force to sterilization of the females in the classes considered and for reasons which do not need enumeration.

A recent writer has asserted that the practice of voluntary sterilization is a horrible crime and so revolting that it should be penalized by statute, but Dr. H. C. Sharp of Indiana, who has performed the operation many times, believes the results salutary in every way. In habitual criminals and in the incurable insane sterilization protects them from themselves as nothing else can, and it is difficult to see how it can have any bad results in those classes.

But compulsory sterilization in these same classes is objectionable upon at least two grounds. It violates a moral prejudice which is well-nigh universal in the human race, and from a purely eugenic standpoint it is not necessary and it practically accomplishes very little. For reasons other than those under consideration here such care of the hopelessly insane and of habitual criminals is necessary, that they can not be a very great menace to the race through their progeny.

The results which have followed compulsory sterilization of idiots and the feeble-minded, and of voluntary vasectomy in habitual criminals in Indiana have been so favorable that it is not improbable that the practice will extend to the other States of the Union. —*Editorial Southern California Practitioner.*

